# Nur transcription factors in stress and addiction

**DOI:** 10.3389/fnmol.2013.00044

**Published:** 2013-12-02

**Authors:** Danae Campos-Melo, Danny Galleguillos, Natalia Sánchez, Katia Gysling, María E. Andrés

**Affiliations:** Nucleus Millennium in Stress and Addiction, Department of Cellular and Molecular Biology, Faculty of Biological Sciences, Pontificia Universidad Católica de ChileSantiago, Chile

**Keywords:** Nurr1, Nur77, Nor1, corticotropin releasing factor, addiction, stress, nuclear receptors, gene expression regulation

## Abstract

The Nur transcription factors Nur77 (NGFI-B, NR4A1), Nurr1 (NR4A2), and Nor-1 (NR4A3) are a sub-family of orphan members of the nuclear receptor superfamily. These transcription factors are products of immediate early genes, whose expression is rapidly and transiently induced in the central nervous system by several types of stimuli. Nur factors are present throughout the hypothalamus-pituitary-adrenal (HPA) axis where are prominently induced in response to stress. Drugs of abuse and stress also induce the expression of Nur factors in nuclei of the motivation/reward circuit of the brain, indicating their participation in the process of drug addiction and in non-hypothalamic responses to stress. Repeated use of addictive drugs and chronic stress induce long-lasting dysregulation of the brain motivation/reward circuit due to reprogramming of gene expression and enduring alterations in neuronal function. Here, we review the data supporting that Nur transcription factors are key players in the molecular basis of the dysregulation of neuronal circuits involved in chronic stress and addiction.

## Introduction

**Figure 1:**
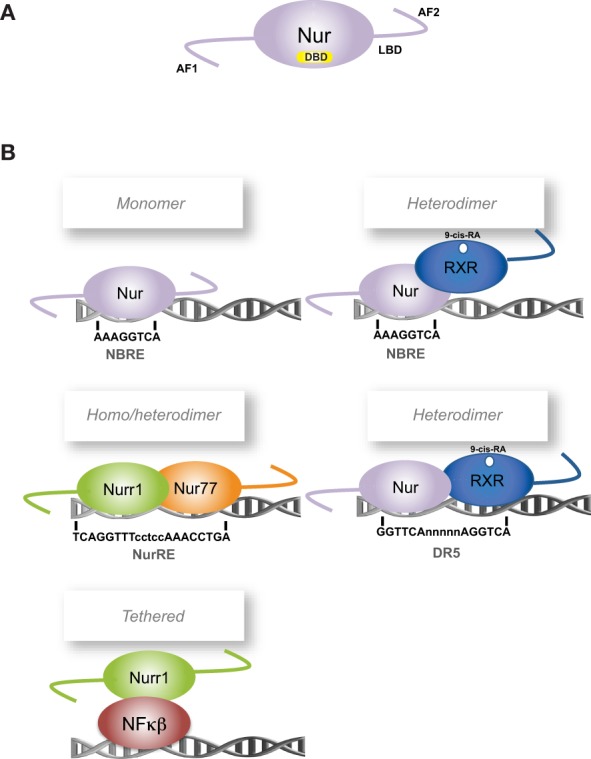
**Scheme of Nur factors structure and DNA-binding elements**. **(A)** Nur factors common structure, AF: activation function domain; DBD: DNA Binding Domain; LBD: Ligand Binding Domain. **(B)** Nur factors DNA binding elements. RXR: retinoic-X-receptor; 9-cis RA: 9-cis retinoic acid.

The transcription factors Nur77 (NGFI-B, NR4A1) (Hazel et al., 1988; Milbrandt, 1988), Nurr1 (NR4A2) (Law et al., 1992) and Nor-1 (NR4A3) (Ohkura et al., 1996) are orphan members of the nuclear receptor superfamily and together conform the Nur subfamily. Nur transcription factors, as members of the nuclear receptor superfamily, share their classic structural organization (Figure 1A) encompassing: (a) a non-conserved N-terminal region containing the transcriptional activation function-1 (AF-1), (b) a conserved DNA binding domain (DBD) located in the middle of the proteins, with 90.2% of amino acid sequence identity among all rat Nur factors, and (c) a moderately conserved C-terminal domain, which encloses the ligand-binding domain (LBD) and the ligand-dependent transcriptional activation function 2 or AF-2 (Giguere, 1999). Although Nur transcription factors have a well-recognized LBD structure, their transcriptional activity is not regulated by endogenous ligands as it is for steroid nuclear receptors (Benoit et al., 2004). Crystallographic studies show that the putative ligand-binding pocket of Nurr1 and Nur77 LBDs are filled with side chains of large hydrophobic amino acids, which keep the LBD in a transcriptionally active conformation (Wang et al., 2003; Flaig et al., 2005). Since the binding of ligands does not trigger the transcriptional activity of Nur factors, changes of their expression levels and post-translational modifications appear keys to regulate their activity.

Nur factors behave as immediate early genes, and as such, their mRNA expression is induced independent of protein synthesis in several cell types by multiple kinds of stimuli (Williams and Lau, 1993; Maruyama et al., 1995; Satoh and Kuroda, 2002; Maxwell and Muscat, 2006). Nur factors show some basal expression in specific nuclei of the rodent brain (Zetterstrom et al., 1996b). However, remarkable fast and high induction has been observed for Nur factors in selected brain nuclei or dissociated neurons after physiological, chemical or toxic stimulation (Chan et al., 1993; Honkaniemi et al., 1994; Jacobs et al., 1994; Umemoto et al., 1994; Svenningsson et al., 1995; Imaki et al., 1996; Svenningsson and Fredholm, 1997; Tang et al., 1997; Xing et al., 1997; Honkaniemi and Sharp, 1999; Brosenitsch and Katz, 2001; Ojeda et al., 2003; Maheux et al., 2005). These data strongly support the notion that Nur factors have homeostatic functions and, functioning as immediate-early gene, can also serve as rapid signaling system.

To exert their function as transcriptional regulators, Nur factors bind to nerve-growth-factor inducible gene B (NGFI-B)-responsive element (NBRE) (A/TAAAGGTCA) (Wilson et al., 1991) as monomers (Figure 1B) (Wilson et al., 1993a; Paulsen et al., 1995), and to Nur-responsive element (NurRE) (inverted repeated of NBRE-related octanucleotide, AAATG/AC/TCA) (Philips et al., 1997) as homodimers or heterodimers between Nur factors (Philips et al., 1997; Maira et al., 1999). Nur77 and Nurr1, but not Nor-1, can also form heterodimers with RXR retinoid receptor (Perlmann and Jansson, 1995; Zetterstrom et al., 1996a). These heterodimers activate transcription through NBRE elements, where the Nur component binds to the NBRE element, or through DR5 elements (two direct repeats of the consensus nuclear receptor binding motif separated by five nucleotides, GGTTCAnnnnnAGGTCA). In the case of DR5 elements, both nuclear receptors bind to the DNA (Perlmann and Jansson, 1995). In addition, Nur factors can regulate transcription indirectly by binding to another transcription factor. For instance, Nurr1 represses transcription of inflammatory genes in microglia indirectly by forming a complex with NF-kB transcription factor (Saijo et al., 2009).

**Figure 2:**
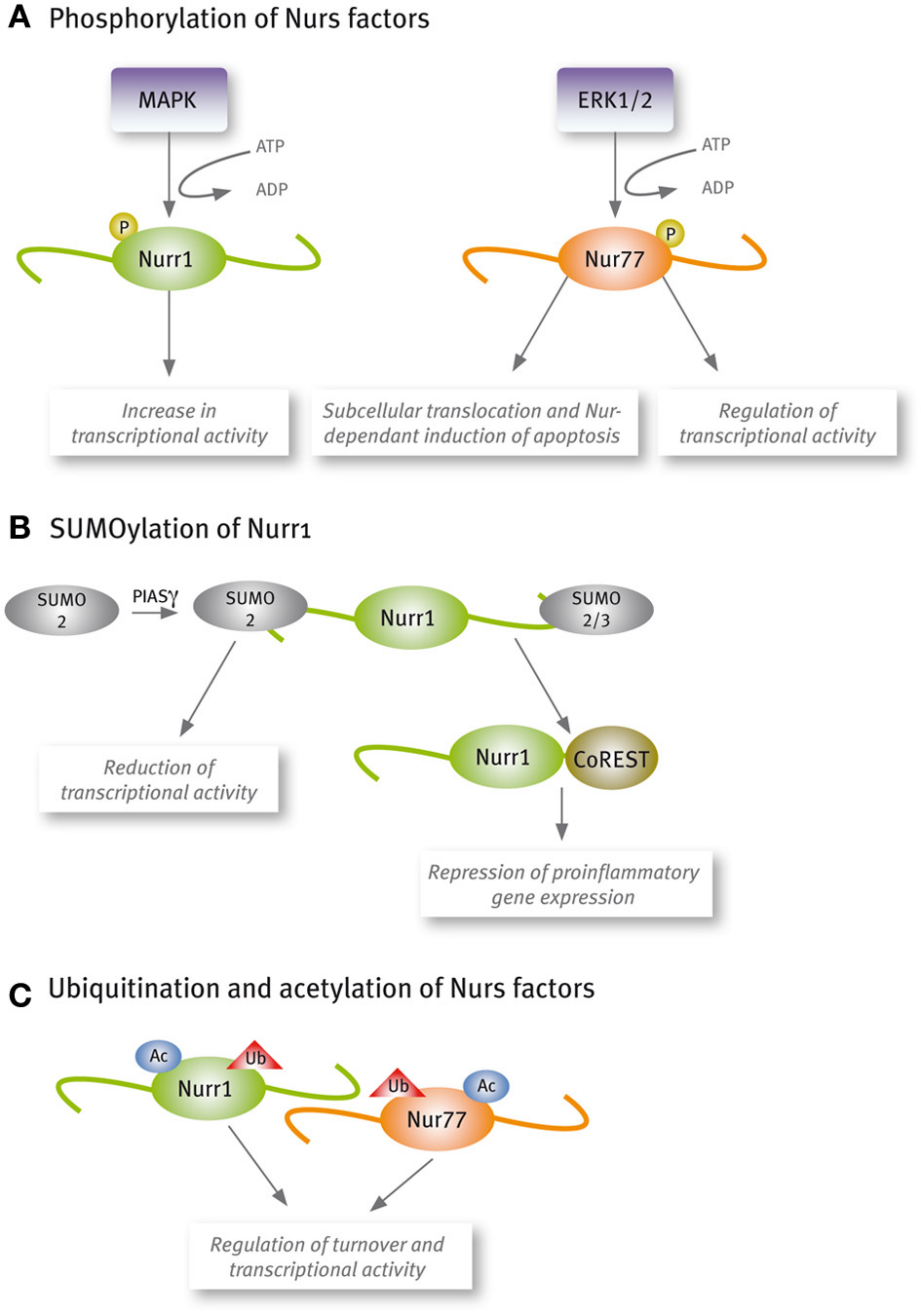
**Nur factors are post-translational modified**. **(A)** Phosphorylation, **(B)** SUMOylation, and **(C)** Acetylation and ubiquitination.

Increasing reports show that Nur factors transcriptional activity is regulated by post-translational modifications (Figure 2). Phosphorylation of Nur77 by extracellular-signal regulated kinase 2 (ERK2) is required for its transcriptional activity in corticotrophs (Kovalovsky et al., 2002). Similarly, Nurr1 phosphorylation by mitogenic-activated protein kinases increases its transcriptional activity (Nordzell et al., 2004; Sacchetti et al., 2006; Zhang et al., 2007). Phosphorylation of Nur factors plays an important role also in their subcellular translocation and Nur-dependent induction of apoptosis (Katagiri et al., 2000; Slagsvold et al., 2002; Wingate et al., 2006). The post-translational modification SUMOylation has appeared as an important regulatory pathway of Nurr1 transcriptional activity (Galleguillos et al., 2004; Saijo et al., 2009; Arredondo et al., 2013). All Nur factors harbor SUMO consensus motifs in their sequences, but only Nurr1 SUMOylation by SUMO2 and SUMO3 has been demonstrated. SUMOylation of the N-terminal domain of Nurr1 with SUMO2 reduces its transcriptional activity in promoters harboring more than one NBRE element (Arredondo et al., 2013). Also, SUMOylation with SUMO2 or SUMO3 of the C-terminal of Nurr1 is required to bind the corepressor CoREST and repress pro-inflammatory gene expression (Saijo et al., 2009). Recent work has shown that Nur factors turnover and transcriptional activity are regulated by acetylation (Kang et al., 2010) and ubiquitination (van Tiel et al., 2012; Alvarez-Castelao et al., 2013).

Nur factors are associated to several functions (Maxwell and Muscat, 2006). Some of the functions are exclusive of one factor, like the essential role of Nurr1 in the induction and maintenance of midbrain dopamine neurons (Zetterstrom et al., 1997; Castillo et al., 1998; Saucedo-Cardenas et al., 1998; Kadkhodaei et al., 2009), and Nor-1 requirement for normal embryonic development (Deyoung et al., 2003), inner ear (Ponnio et al., 2002), and hippocampus development (Ponnio and Conneely, 2004). Increasing evidence indicates that all Nur factors play significant roles in inflammatory (McMorrow and Murphy, 2011; van Tiel and de Vries, 2012) and oncogenic processes (Mohan et al., 2012). Increased expression levels of Nur factors have been observed in several types of cancer cell lines and tumors, although the opposite has also been reported. Collected data suggest that Nur77 behaves as a pro-oncogenic factor (Lee et al., 2011). However, the double knockout of Nur77 and Nor-1 induced a fatal acute myeloid leukemia in mice (Mullican et al., 2007), indicating that these nuclear receptors may also play a role as tumor suppressors (Mullican et al., 2007). The apparent controversy or dual role of Nur factors as tumor suppressors and/or pro-oncogenic factors could be explained by a dual role in transcription, behaving as transcriptional activators or repressors. Since Nur factors are transcriptionally active in their native form, less attention has been paid to their role as potential transcriptional repressors. Interestingly, there are several reports showing the interaction of Nur factors with transcriptional corepressors. For example, we showed that PIASγ interacts and represses Nurr1-dependent transcriptional activity (Galleguillos et al., 2004). Nurr1 also interacts with the transcriptional repressors SMRT (Lammi et al., 2008; Jacobs et al., 2009) and CoREST (Saijo et al., 2009). The interaction with SMRT maintains Nurr1 in a transcriptional repressive complex impeding the induction of its dopaminergic target genes (Jacobs et al., 2009). It has also been shown the interaction between Nur77 and SMRT (Sohn et al., 2001). Regarding the role of Nur77 and Nor-1 repressing the expression of target genes, it was shown that abrogation of these transcription factors correlates with an increased expression of MYC oncogene (Boudreaux et al., 2012). In addition, it was demonstrated that MYC is a direct target gene of Nur77/Nor-1, whose expression was strongly repressed in a Nur DNA-binding dependent way (Boudreaux et al., 2012). Nur77 and Nor-1 play also an important role triggering apoptosis in several cell types. Interestingly, this effect is due to the translocation of Nur77/Nor-1 from the nuclei to the mitochondria, where Nur77 triggers cytochrome c release and apoptosis (Li et al., 2000). Through this mechanism, Nur77 and Nor-1 play a central function in the clonal deletion of autoreactive thymocytes (Sohn et al., 2007). Nur77 and Nor-1 colocalize in several cell types, including CNS neurons, and apparently they replace each other in some functions. This colocalization of Nur77 and Nor-1 explain the lack of deleterious effects in the single knockout mice, while lack of both induces a catastrophic deregulation in the immune system. Similarly, colocalization of Nur77 with Nor-1 in the CNS may explain an apparent lack of strong effect of each knockout in the stress and rewarding systems of the brain.

## Nur transcription factors and dopaminergic transmission

**Figure 3:**
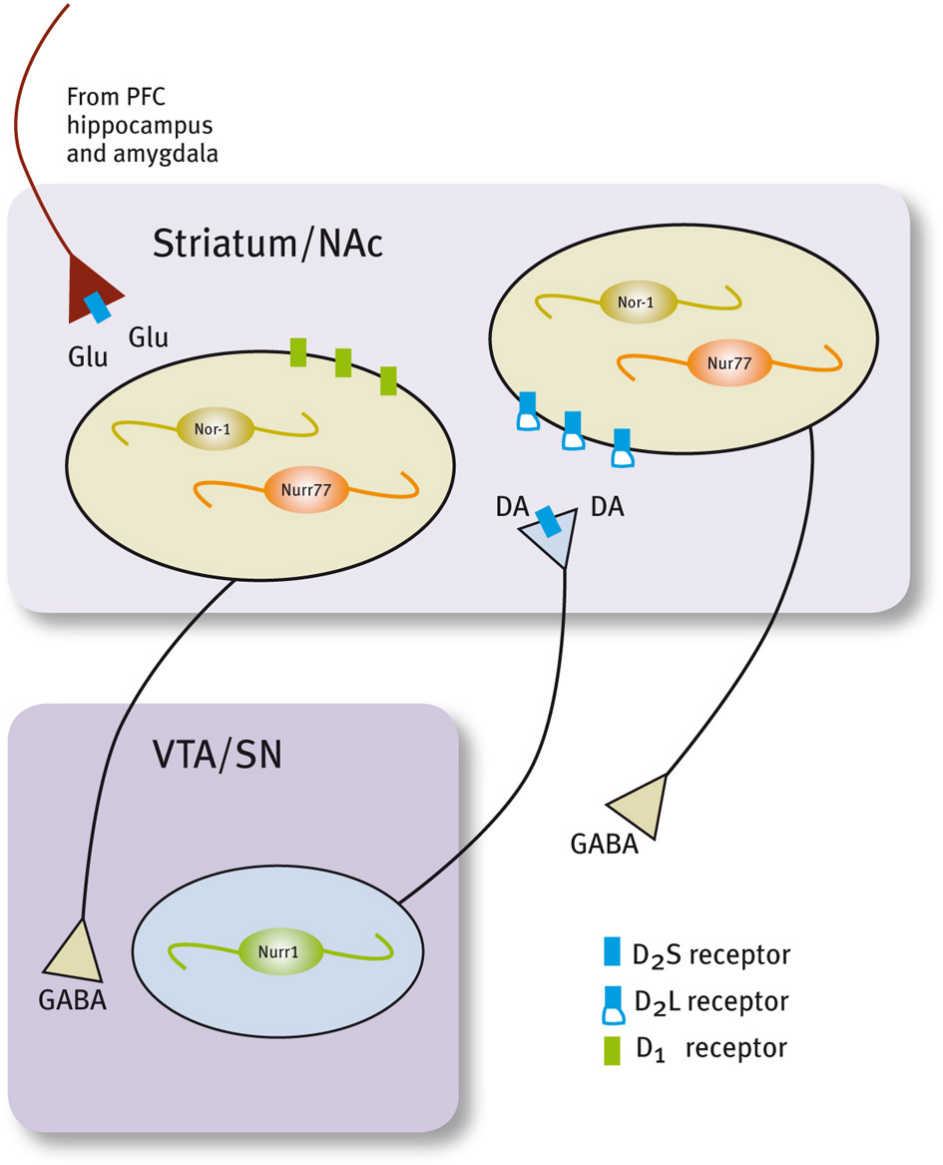
**Expression of Nur factors in the in the motivation/reward circuit**. Nurr1 is expressed in dopamine neurons of the SN/VTA and Nur77 and Nor-1 are expressed in dopamine-receptive GABAergic neurons of the striatum/NAc. GABAergic projecting neurons in the striatum/NAc are segregated in two populations that express either D1 or D2 receptors (Valjent et al., 2009).

Histological and neurochemical evidence supports the idea that Nur transcription factors are closely associated with dopamine neurotransmission (Figure 3, Table 1). Nur77 and Nor-1 are expressed in neurons of the striatum, the nucleus accumbens (NAc) and the prefrontal cortex (PFC) (Xiao et al., 1996; Zetterstrom et al., 1996b; Gervais et al., 1999; Werme et al., 2000a,b; Davis and Puhl, 2011), all target nuclei of dopaminergic neurons originated in the ventral midbrain. In these nuclei, a strong control of Nur77 and Nor-1 expression is induced by the stimulation of the dopamine neurons (Chergui et al., 1997); by the administration of dopamine D2 receptor agonists and antagonists (Gervais et al., 1999; Beaudry et al., 2000; Werme et al., 2000b; Langlois et al., 2001; Maheux et al., 2005); by drugs of abuse (Werme et al., 2000a; St-Hilaire et al., 2003b) and after dopamine denervation. Collected data indicate that Nur77 expression is under a tonic inhibitory control exerted by physiological dopamine basal levels through D2 receptor. Acute administration of dopamine D2 agonists decreases Nur77 mRNA levels in the striatum (Gervais et al., 1999), while the opposite is observed after acute dopamine D2 antagonist administration in the striatum, NAc and PFC (Beaudry et al., 2000; Langlois et al., 2001; Maheux et al., 2005). Interestingly, D2 antagonist-dependent induction of Nur77 expression in the striatum and NAc core is preserved after chronic treatment with D2 ligands (Beaudry et al., 2000; Werme et al., 2000b; Mahmoudi et al., 2009, 2013), suggesting that this transcription factor mediates adaptive changes to long-term repetitive dopamine variations (Levesque and Rouillard, 2007).

**Table 1:** **Nur transcription factors and dopaminergic neurotransmission**.

**Nur factor**	**Expression**	**Effect of DA agonists, antagonists, psychostimulants, and 6-OHDA**		**KO characteristics**
Nur77	High: Striatum, NAc, PFC, olfactory bulb and tubercle, cortex, hippocampal formation, hypothalamus and amygdala^a^^−^^e^	D1 agonist:	No effects in striatum^f^^,^ ^g^	D2R increases in dorsal striatum^m^^,^ ^n^
		D2 agonist:	mRNA down- regulated in striatum^f^^,^ ^g^	Alterations in the expression of NT and ENK^m^^,^ ^o^
	Low: VTA, SN^a^^−^^e^	D2 antagonist:	mRNA up-regulated in striatum, PFC,	Increased locomotor activity^n^
			NAc, VTA, and SN^c^^,^ ^h^^,^ ^i^^,^ ^j^^,^ ^p^	Increased sensitivity to low dose of D2 agonist^n^
		Psychostimulants: (amphetamine, cocaine, and others)	mRNA up-regulated in striatum and NAc^e^^,^ ^k^^,^ ^l^	Increased DOPAC in SN and VTA^n^
		D1 and D2 agonists:	mRNA up-regulated in striatum^f^	Decreased cataleptic response after D2R antagonist^m^
		6-OHDA in striatum:	mRNA up-regulated in striatum^f^	
				Decreased COMT expression^n^
			L-DOPA treatment: complex regulation in striatum^f^	
Nor-1	High: Striatum, NAc, PFC, olfactory bulb and tubercle, cortex, hippocampal formation, hypothalamus and amygdala^a^^−^^e^	D2 Antagonist:	mRNA up-regulated in striatum, NAc, PFC, VTA and SN^h^^,^ ^p^	Not viable embryos. Embryos die at early stages^q^
		Psychostimulants: (amphetamine, cocaine and others)	mRNA up-regulated in NAc, striatum and cortex^e^^,^ ^l^	
	Low: VTA, SN^a^^−^^e^			
Nurr1	High: VTA, SN^a^^,^ ^b^	Psychostimulants in drug abusers: (amphetamine, cocaine and others)	mRNA down-regulated in SN and VTA^q^^,^ ^r^	Agenesis DA neurons^t^
	Low: cortex, hippocampal formation, olfactory bulb, hypothalamus^a^^,^ ^b^			
		6-OHDA in striatum:	mRNA up-regulated in SN^s^	

*DA: dopamine; 6-OHDA: 6-hydroxydopamine; L-DOPA: 3,4-dihydroxyphenylalanine; DOPAC: dihydroxyphenylacetic acid; COMT: catechol-O-methyltransferase; ENK: enkephalin; NT: neurotensin*.

a Gofflot et al., 2007

b Zetterstrom et al., 1996a,b

c Beaudry et al., 2000

^d^Ponnio and Conneely, 2004

e Werme et al., 2000a,b

f St-Hilaire et al., 2003a

g Gervais et al., 1999

h Maheux et al., 2005

i Langlois et al., 2001

j Maheux et al., 2012

k St-Hilaire et al., 2003b

l Krasnova et al., 2011

m Ethier et al., 2004

n Gilbert et al., 2006

o St-Hilaire et al., 2006

p Maheux et al., 2012

q Nielsen et al., 2008

r Horvath et al., 2007

s Ojeda et al., 2003

t *Zetterstrom et al., 1997; Castillo et al., 1998; Saucedo-Cardenas et al., 1998*.

Studies using dopamine denervation and Nur77 knockout mice have been instrumental to reveal the role of Nur77 as a master factor for adaptations induced by dopaminergic neurotransmission changes. Full lesion of dopamine nigro-striatal pathway and D2 antagonist administration produce a significant up-regulation of both Nur77 and enkephalin mRNA in enkephalin positive neurons of the striatum (Beaudry et al., 2000; St-Hilaire et al., 2003a, 2005). Enkephalin up-regulation induced by D2 antagonists or by dopamine denervation is severely impaired in Nur77 knockout mice (Ethier et al., 2004; St-Hilaire et al., 2006). Moreover, the normalization of enkephalin levels induced by L-DOPA treatment of dopamine denervated animals is not observed in Nur77 knockout mice (St-Hilaire et al., 2006), indicating that Nur77 is essential for this adaptive effect. Interestingly, a recent publication suggests that the D2 control over Nur77 expression in the striatum is due to presynaptic D2 receptors located on glutamatergic terminals coming from the cortex (Maheux et al., 2012). Metabotropic mGlu5 receptor antagonist suppressed D2 antagonist-induced Nur77 expression (Maheux et al., 2012), indicating that glutamate positively controls Nur77 expression in the striatum. These data show that Nur77 may play an integrative role of multiple signaling in the striatum.

mRNA levels of Nur77 and Nor-1 are extremely low or absent in adult midbrain dopaminergic neurons of substantia nigra (SN) and ventral tegmental area (VTA) in basal conditions (Zetterstrom et al., 1996a,b). However, current data indicate that Nur77 may play an important role regulating dopamine biochemical homeostasis in these nuclei. Nur77-deficient mice show an increase in dopamine D2 receptors in dorsolateral striatum; enhanced spontaneous locomotor activity; greater sensitivity to dopamine D2 receptor agonists and higher levels of DOPAC relative to the wild-type (Gilbert et al., 2006). Remarkably, Nur77 and Nor-1 expression increases significantly in midbrain dopamine neurons after the administration of dopamine D2 receptor antagonists (Maheux et al., 2005).

Unlike Nur77 and Nor-1, Nurr1 is expressed under basal physiological conditions in dopamine neurons of SN and VTA (Xiao et al., 1996; Zetterstrom et al., 1996a; Backman et al., 1999; Ojeda et al., 2003). Strong evidence indicates that Nurr1 is essential for the development of these neurons (Zetterstrom et al., 1997; Castillo et al., 1998; Saucedo-Cardenas et al., 1998). More recently, it was shown that Nurr1 is also critical to maintain midbrain dopamine neurons in the adult (Kadkhodaei et al., 2009). During development, dopamine midbrain precursors do not differentiate into dopaminergic phenotype in the Nurr1 null mice (Zetterstrom et al., 1997; Castillo et al., 1998; Saucedo-Cardenas et al., 1998). Dopaminergic markers like tyrosine hydroxylase (TH), the dopamine transporter (DAT), the vesicular monoamine transporter (VMAT2) and the tyrosine kinase receptor RET (Wallen et al., 2001) are not expressed in the ventral mesencephalon of Nurr1 null mice at birth (reviewed in Smidt et al., 2003; Perlmann and Wallen-Mackenzie, 2004). On the other hand, a progressive decline in the expression of the same genes in the SN and VTA is observed when Nurr1 is ablated in adults (Kadkhodaei et al., 2009, 2013; Galleguillos et al., 2010). Interestingly, it was recently shown that Nurr1 regulates the expression of a set of mitochondria genes (Kadkhodaei et al., 2013). Whether, the role of Nurr1 keeping dopamine neuron fully functional during adulthood and aging is related to the control of genes of the phenotype or mitochondrial genes or both requires further evidence.

The accumulated evidence indicate that Nurr1 regulates the transcription of dopamine phenotype genes (Sakurada et al., 1999; Iwawaki et al., 2000; Sacchetti et al., 2001; Hermanson et al., 2003; Kim et al., 2003; Smits et al., 2003; Galleguillos et al., 2010). Studies in Nurr1-deficient mice (±) indicate that the amount of Nurr1 is important to keep homeostasis during the life of dopamine neurons (Le et al., 1999; Jiang et al., 2005; Eells et al., 2006; Zhang et al., 2012). For instance, aged Nurr1 (±) mice have a significant decrease in rotarod performance and locomotor activity, a motor impairment analogous to Parkinson's disease associated with decreased dopamine levels in the striatum (Jiang et al., 2005). Recently, it was shown that a decreased number of TH positive cells in SN, observed in aged Nurr1 (±) mice, correlates with decreased dopamine release in the striatum (Zhang et al., 2012). Similarly, we showed that inducing a 50% decrease of Nurr1 expression in the SN of adult rats results in a significant decrease of dopamine extracellular levels in the striatum associated with decreased expression of the tyrosine kinase receptor RET (Galleguillos et al., 2010). Nurr1-deficient mice (±) also show some symptoms related to schizophrenia, such as hyperactivity in a novel environment, deficiency in the retention of emotional memory and increased response to swim stress; all symptoms associated with dysfunctions in dopamine neurotransmission (Rojas et al., 2007; Vuillermot et al., 2011). Thus, these data show that Nurr1 controls the expression of dopamine phenotype genes during adulthood. In addition, the data show that survival pathways, which are stressed during aging, require a stronger Nurr1 signaling, that cannot be achieved in Nurr1 heterozygous mice. Remarkably, the amount of Nurr1 protein levels seems to play a significant role controlling the expression of specific sets of target genes. Indeed, the group of Bannon (Johnson et al., 2011), showed that different set of genes are controlled by lower vs. higher Nurr1 level.

How Nurr1 is regulated by dopamine levels and adjusts the expression of target genes accordingly? Accumulated data indicates that Nurr1 expression depends on dopamine signaling, mainly through D2 receptors. D2 receptors are located in soma and dendrites, to regulate firing rate of neurons and presynaptically in axons to regulate dopamine synthesis and release. Drugs of abuse like cocaine decrease Nurr1 in mesencephalon (Bannon et al., 2002, 2004). D2 receptor knockout mice have increased Nurr1 expression in midbrain dopamine neurons (Tseng et al., 2000) and the loss of dopamine in rat striatum induced by 6-hydroxydopamine generates a rapid increase of Nurr1 expression in dopamine neurons of the SN (Ojeda et al., 2003). These data show that dopamine extracellular levels influence the expression of Nurr1 in the SN and VTA. Recent evidence indicates that neuronal firing regulates differentially the expression of Nurr1 and Nur77 in dopamine neurons (Eells et al., 2012). A normal flow of impulses maintains basal expression of Nurr1. Surprisingly, Nur77 was induced with a lower D2 autoreceptor activation in the VTA (Eells et al., 2012). Recently, it was shown that D2 receptors located postsynaptically in GABA projecting neurons of the striatum also regulate dopamine release (Anzalone et al., 2012). Nurr1 and/or Nur77 could be the targets of such a feedback mechanism regulating dopamine homeostasis. The recent findings of Nur77 controlling dopaminergic homeostasis raises several questions regarding the specific role of each Nur factor in dopaminergic gene expression: Are Nur factors redundant or they play particular roles in the adaptation and survival processes of midbrain dopamine neurons during adulthood and aging?

## Nur transcription factors during the stress response

In response to stressful stimuli, secretory neurons of the paraventricular nucleus (PVN) discharge corticotropin-releasing factor (CRF) that in turn increases both the secretion of adrenocorticotrophin hormone (ACTH) and the transcription of its precursor, the proopiomelanocortin (POMC) gene, in the anterior pituitary. ACTH stimulates the release of glucocorticoids (GCs) and the transcription of genes encoding several steroidogenic enzymes in the adrenal gland. GCs exert diverse effects on target tissues to mobilize energy for the body to deal with the stressor, and also exert a negative feedback through the inhibition of the synthesis and secretion of CRF and POMC (Keller-Wood and Dallman, 1984; Sawchenko and Swanson, 1985; Antoni, 1986; Swanson and Simmons, 1989).

**Figure 4:**
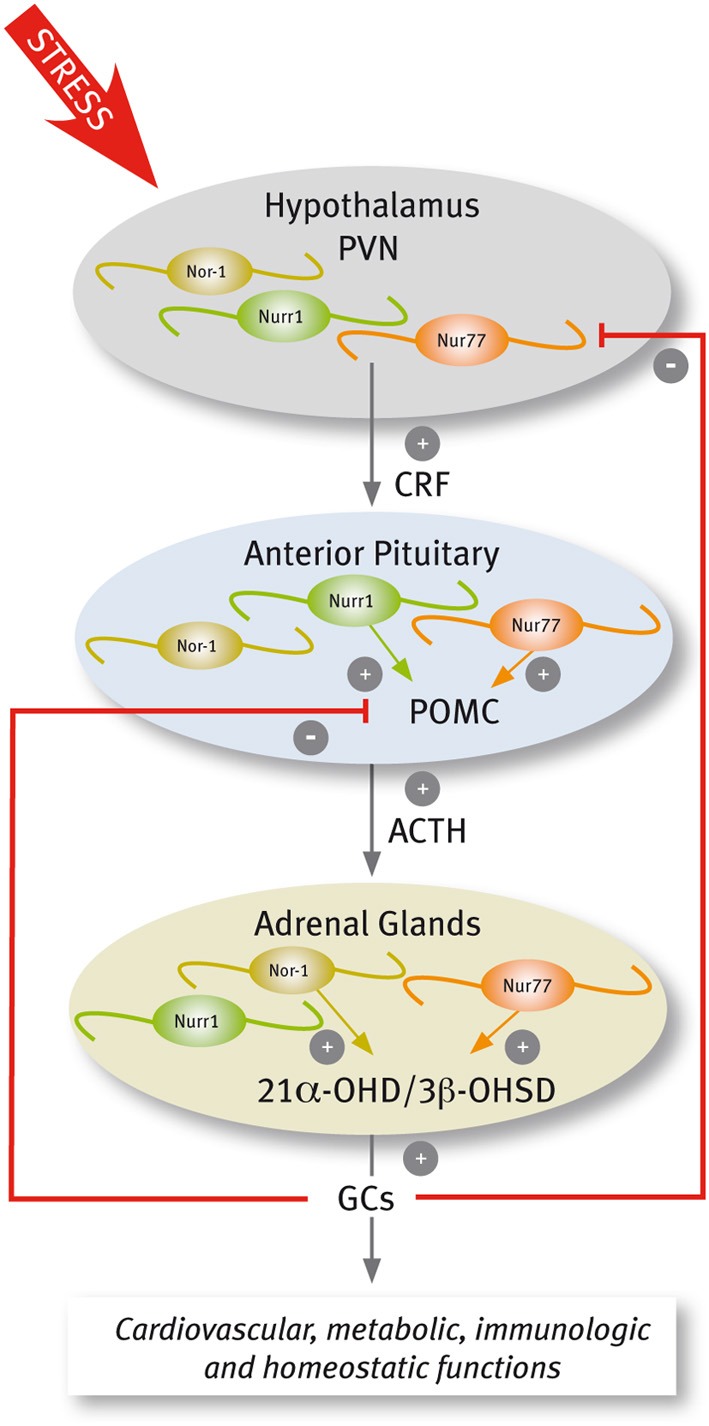
**Scheme of Nur factors expression in the HPA axis**.

Several lines of evidence indicate that Nur transcription factors play a prominent role in adaptive responses to stress, regulating the transcription of target genes in the hypothalamus-pituitary-adrenal (HPA) axis (Figure 4). The evidence indicates that this nuclear receptor subfamily regulates the expression of CRF and POMC in the PVN and pituitary, respectively (Murphy and Conneely, 1997; Drouin et al., 1998). In the adrenal glands, Nur77 and Nor-1 also regulate the expression of steroid-21α-hydroxylase and 3-β-hydroxysteroid dehydrogenase, both enzymes essential for the production of GCs (Figure 4) (Wilson et al., 1993b; Fernandez et al., 2000; Bassett et al., 2004).

A complex signaling cascade involving Nur factors is required for POMC regulation. The group of Eduardo Arzt (Kovalovsky et al., 2002) showed that in AtT-20 corticotrophs, CRF induces both Nur77 and Nurr1 expression resulting in POMC transcription through a NurRE site in its promoter. While CRF-mediated induction of Nur factors requires protein kinase A (PKA) and cAMP signaling, POMC transcriptional induction depends further on MAPK activation. Nur77-dependent induction of POMC transcription required the phosphorylation of Nur77 by MAPK (Kovalovsky et al., 2002). Concordantly, cAMP/PKA signaling enhances DNA binding activity of Nur dimers, but not monomers, and the recruitment of transcriptional coactivators through the AF-1 domain of Nur factors (Maira et al., 2003). Nur transcription factors are also involved in the ending of the stress response. The effect of Nur transcription factors on POMC promoter is antagonized by GCs (Philips et al., 1997; Drouin et al., 1998; Martens et al., 2005; Carpentier et al., 2008). The NurRE element of the POMC gene, which binds Nur dimers, is the target of the repressive effect of GCs. GC receptors bound to ligands directly interact with Nur factors inducing the transrepression of POMC (Martens et al., 2005).

Less is known about the role of Nur factors regulating CRF in the PVN. While Nurr1 is expressed constitutively in the PVN (Saucedo-Cardenas and Conneely, 1996), Nur77 is transiently induced by different types of stressful stimuli (Chan et al., 1993; Honkaniemi et al., 1994; Imaki et al., 1996; Kawasaki et al., 2005), and by central administration of CRF (Parkes et al., 1993). Interestingly, an increased expression of Nur77 occurs in the PVN of virgin and lactating females, but not in pregnant mice stressed either by exposure to a novel environment or forced swimming (Douglas et al., 2003), suggesting a role for sex hormones regulating Nur factors action. In 1997, Murphy and Conneely showed that Nurr1 and Nur77 bind and transactivate the CRF gene (Murphy and Conneely, 1997). The CRF gene promoter harbors a conserved NBRE element (Murphy et al., 1996; Yao and Denver, 2007; Yao et al., 2007), which is required for Nur77-dependent inductive effect of reporters driven by the CRF promoter (Murphy and Conneely, 1997). More recently, Stroth et al. (2011) showed that up-regulation of mRNAs encoding CRF and Nur factors in the PVN during stress depends on Pituitary Adenylate Cyclase Activating Polypeptide (PACAP). Indeed, not only CRF, but also restraint-induced expression of Nur77 and Nurr1 decreases in the pituitary gland of PACAP-deficient mice (Stroth et al., 2011), suggesting a reciprocal regulation between Nur factors and CRF. Normal function of the HPA axis in Nur77 null mutant mice indicates redundancy of Nur factors. Nurr1 mRNA increases in the adrenal gland of Nur77 null mice (Crawford et al., 1995). In addition, Nor-1 and Nur77 play similar, albeit distinct roles in the HPA axis. Similar to Nur77, Nor-1 activates the expression of the gene encoding steroid-21α-hydroxylase (Wilson et al., 1993b; Fernandez et al., 2000) through NBRE elements. In contrast, DNA binding experiments of Nor-1 to NurRE element suggest that Nor-1 is not an efficient substitute of Nur77 activation of POMC gene expression in the pituitary gland (Fernandez et al., 2000).

In addition to its crucial role in the activation of the HPA axis, CRF functions as a neurotransmitter/neuromodulator coordinating extra-hypothalamic aspects of the stress response (Aguilera, 1998; Ziegler and Herman, 2000; Herman et al., 2002). The extra-hypothalamic aspects of the stress response are mediated by interconnected nuclei of the amygdala and the bed nucleus of the stria terminalis (BNST) (Walker et al., 2003). The BNST is the primary integrative center of excitatory and inhibitory inputs regulating the HPA axis during stress (Forray and Gysling, 2004), and mediates anxiety-like behavior resulting of extended threat (Walker et al., 2009). The expression of Nur transcription factors increases in the limbic nuclei associated to the extra-hypothalamic aspects of the stress response, suggesting that Nur factors could also underlie enduring changes induced by chronic stress. For instance, Nor-1 is increased in the somatosensory cortex and amygdala when exposed to novelty stress (Sun et al., 2007). Predator stress increases Nur77 mRNA in prelimbic, infralimbic and, ventral and lateral orbital prefrontal cortexes (Schiltz et al., 2007). Nurr1 expression increases while TH decreases at postnatal day 7 in the VTA of prenatally stressed offspring, which suggest a possible compensatory mechanism that may play Nurr1 to counteract the observed reduction of dopamine levels (Katunar et al., 2009, 2010). In addition, dopamine content decreases within the PFC and midbrain of rats subjected to forced swim test, meanwhile Nurr1 expression increases in the same brain areas (Rojas et al., 2010) supporting a role for Nurr1 counteracting the decrease of dopamine content. Intriguingly, Eells et al. (2002) showed that Nurr1 (±) mice displayed significantly greater locomotor activity in response to mild stress that correlated with lower dopamine content in mesolimbic and mesocortical circuits (Eells et al., 2002). Taken together the data indicate that Nur factors expression is modulated in limbic circuit during the response to stress. It is unknown what are the genes induced or repressed by Nur factors in the nuclei associated to the extra-hypothalamic aspects of the stress response. One possible target is CRF.

Repeated immobilization stress and chronic mild stress induce CRF expression in the BNST (Stout et al., 2000; Santibanez et al., 2006). We reported that cells expressing CRF in the BNST also express Nur77 (Campos-Melo et al., 2011), even though the expression of Nur77 is wider than CRF in this nucleus. Nur77 expression increases significantly in the dorso-lateral and ventro-medial subdivisions of the BNST after acute and repeated immobilization stress (Campos-Melo et al., 2011), same areas where CRF expression increases after repeated immobilization stress (Santibanez et al., 2006). Several years ago, it was shown that the intracerebroventricular injection of CRF increases CRF expression in the PVN (Parkes et al., 1993). A CRF-dependent induction of CRF could be a mechanism of maintaining CRF expression in brain nuclei associated to chronic stress-induced anxiety and depression (Stenzel-Poore et al., 1994; Pelton et al., 1997; Yao and Denver, 2007). In support of this suggestion (Parkes et al., 1993), immunohistochemical data show that CRF neurons are innervated by CRF axons in PVN and amygdala (Moga et al., 1989; Silverman et al., 1989; Moga and Saper, 1994). Similarly, in BNST, positive CRF terminals also innervate CRF neurons (Sakanaka et al., 1986; Veinante et al., 1997). Since CRF induces the expression of Nur77 (Kovalovsky et al., 2002) and Nur77 also is able to induce CRF expression (Murphy and Conneely, 1997), it is possible that Nur77 could mediate the vicious cycle of CRF-dependent CRF induction in the PVN and limbic nuclei, during chronic stress; an hypothesis that requires further investigation.

## Functional role of Nur transcription factors in the addiction process. protectors or instigators?

The motivation/reward circuit has its roots in dopamine neurons located in the VTA, which send afferences to the NAc, BNST, septum, amygdala, and PFC. Acute administration of drugs of abuse, which increase dopamine release (Di Chiara and Imperato, 1988), induce the expression of Nur77 and Nor-1 in nuclei of the motivation/reward circuit (Table 1) (Werme et al., 2000a; St-Hilaire et al., 2003b; Krasnova et al., 2011). Nur77 and Nor-1 are up-regulated in the NAc, striatum, and cortex after acute administration of cocaine and morphine (Werme et al., 2000a). Similarly, acute administration of methamphetamine up-regulates the expression of Nur77 in cortex, striatum and NAc core; and of Nurr1 in the cortex and VTA. Pretreatment with a selective antagonist of D1/D5 dopamine receptors prevents methamphetamine-induced expression of both Nur77 and Nurr1 mRNA, supporting that dopamine-mediated signaling regulates Nur transcription factors expression (Akiyama et al., 2008). In concordance, the simultaneous administration of D1 and D2 agonists increases Nur77 expression in the striatum (St-Hilaire et al., 2003b). However, D1 agonists administered alone do not modify Nur77 expression in the striatum (St-Hilaire et al., 2003a). As analyzed before, substantial data indicate that dopamine D2 antagonists increase, while D2 agonists decrease Nur77 and Nor-1 expression in the striatum (Beaudry et al., 2000; Werme et al., 2000a,b; Langlois et al., 2001; Maheux et al., 2005, 2012; St-Hilaire et al., 2005). How could this paradox be explained? Taken together the available evidence suggests that drugs of abuse would require another neurotransmitter system, besides dopamine, in order to induce Nur77 in GABA projecting neurons of the striatum. Nur77 induction by D2 antagonists in GABA projecting neurons depends on glutamate signaling through mGlu5 receptors (Maheux et al., 2012). Maheux et al. (2012) showed that ablation of the long isoform of dopamine D2 receptors, located post-synaptically do not prevent D2 antagonists-dependent induction of Nur77, indicating that the effect is presynaptic, where the short isoform of the D2 receptor is present. Presynaptic D2 receptors are located in dopaminergic and glutamatergic axons in the striatum. This result was further supported by showing that interrupting glutamate neurotransmission to the striatum by cortex lesion prevented the increase of Nur77 expression induced by D2 antagonists (Maheux et al., 2012). It is tempting to suggest that similarly, drugs of abuse-dependent induction of Nur77 in the striatum and NAc depends, besides dopamine, on glutamate neurotransmission. Dopamine- and glutamate-dependent induction of Nur77 supports a role for Nur77 integrating pre and postsynaptic information in striatal GABA projecting neurons.

The development of compulsive running, associated with a high risk of addictive behavior, correlates with lower Nur77 and Nor1 expression in several nuclei of the motivation/reward circuit, in the addiction-prone Lewis rat strain compared with the less-addiction prone Fisher rats which do not develop compulsive running (Werme et al., 1999). Accordingly, Nur77 null mice show increased locomotor activity, but a similar locomotor sensitization than wild type mice after repeated amphetamine administration (Bourhis et al., 2009). Interestingly, the blockade of amphetamine-induced locomotor sensitization by an RXR antagonist is abolished in Nur77 null mice (Bourhis et al., 2009). Together the data suggest that Nur77 regulates addiction-prone phenotype and sensitization by different mechanisms. The available information regarding Nurr1 protecting or facilitating addictive behaviors is unclear. In one study it was shown that Nurr1 (±) mice do not develop compulsive running behavior and high ethanol consumption compared to wild type mice (Werme et al., 2003). In another study it was shown that Nurr1 (±) mice have an increased basal locomotor activity and augmented locomotor response to acute methamphetamine administration (Backman et al., 2003). Mice genetic background, behavioral protocols, among other parameters, may influence these observations. Remarkably, in a recent work it was shown that ablation of the histone deacetylase HDAC3 in the NAc facilitates condition-place preference induced by cocaine (Rogge et al., 2013). This effect was correlated with increased Nurr1 expression in this nucleus, supporting a role for Nurr1 facilitating addiction behaviors (Rogge et al., 2013).

It has been proposed that the persistent behavioral and cognitive effects of chronic intake of drugs of abuse depend on new programs of gene expression triggered by immediate-early genes. Tolerance and sensitization of Nur factors expression in nuclei of the motivation/reward and stress brain circuits suggest that these early genes play a signaling role in the plastic changes underlying long-term adaptations. Studies in cocaine abusers showed a reduction of Nurr1 expression in SN neurons (Nielsen et al., 2008). This decreased expression of Nurr1 correlates with a reduction of DAT expression in the same neurons (Bannon et al., 2002). Similarly, rats chronically treated with cocaine show a down-regulation of the expression of Nurr1 mRNA and protein in the ventral midbrain (Leo et al., 2007). Additionally, chronic use of heroin decreases Nurr1 mRNA to a greater extent with age in the paranigral nucleus of the VTA (Horvath et al., 2007). Nurr1 expression decreases after chronic intake of drugs of abuse, could be an adaptive change to excessive dopamine stimulation, and also could be indicative of Nurr1 role adjusting the expression of dopamine target genes during the addiction process. In this regard, it was shown that an acute methamphetamine challenge to animals pretreated with methamphetamine causes a further decrease in Nurr1 mRNA levels (McCoy et al., 2011), indicating that the signaling system regulating Nurr1 expression adapts to new parameters.

Opposing to a tolerance effect observed for Nurr1; Nur77 seems to adapt to dopamine changes and its levels are still inducible after chronic drug intake. For instance, it has been shown that Nur77 expression increases in the frontal cortex of rats after 10 days of cocaine self-administration (Freeman et al., 2002b) or 14 days in a binge model of cocaine administration (Freeman et al., 2002a), and in the dorsal striatum after 7 days of cocaine self-administration (Lynch et al., 2008). On other hand, it was shown that the expression of several early-genes, including Nur factors, is no longer induced in the striatum after chronic exposure to methamphetamine (McCoy et al., 2011). It is noteworthy that Nor-1 is still significantly induced in the striatum of methamphetamine chronically-treated rats, indicating the capacity of the system to respond in new settings.

Chronic stress also triggers tolerance and sensitization of Nur factors expression. In the PVN, it has been shown that Nur77 is no longer induced after chronic stress stimuli (Umemoto et al., 1994, 1997). In contrast, in the ventral region of the BNST, we observed a higher number of cells expressing Nur77 after repeated immobilization stress compared with acute stress (Campos-Melo et al., 2011). Interestingly, the data of the group of Marta Antonelli (Katunar et al., 2010) suggest that Nurr1 may be the transcription factor setting up the new parameters of the dopamine system after prenatal stress. Using prenatal restraint stress, they showed that Nurr1 is permanently higher in the VTA, but not in the SN, in the offspring of stressed mothers. This increment of Nurr1 expression in the VTA correlates with several changes in the motivation/reward dopamine system that persist to adulthood (Baier et al., 2012). An exciting work indicates that Nur factors integrate stress and drug addiction signaling. Postweaning isolation causes elevation in amphetamine-induced dopamine overflow in Nurr1 (−/+) mice, but a reduction in (+/+) mice (Moore et al., 2008). These data demonstrate that a deletion of a single allele of Nurr1, which produced only subtle phenotypic changes, when coupled with a developmental stressor such as postweaning isolation, can dramatically alter mesoaccumbens dopamine neurotransmission (Moore et al., 2008).

## Conclusions

Chronic stress plays a primary role in the origin of several brain pathologies such as anxiety and depression (McCormick and Green, 2013), and facilitates and perpetuates drug addiction (Koob, 2008; Sinha, 2008). Chronic stress and repeated use of addictive drugs induce long-lasting alterations of the motivation/reward circuit and HPA axis (Koob and Le Moal, 2001). The evidence presented points to Nur transcription factors as orchestrators of the molecular bases of the reorganization of these circuits under stressful stimuli and exposure to addictive drugs, since their expression is fast, transient and strongly regulated by dopamine, glutamate, and CRF in the nuclei of the motivation/reward circuit and HPA axis. The features of Nur factors as early genes and orphan nuclear receptors allow them to integrate and transmit fast responses to incoming neurotransmitter signals in neurons. The transient nature of the changes in Nur factor levels suggest that they can re-program the expression of target genes in response to acute and chronic dopamine changes by adjusting their inducibility to the new conditions, as occurring during chronic stress or after repeated exposure to drugs of abuse. Finally, the localization of Nurr1 in dopamine neurons and Nur77/Nor-1 in dopamine-receptive neurons, positions them to translate the dopaminergic information simultaneously to the genome of pre and post-synaptic neurons, allowing an integrative signaling of the motivation/reward circuit. Identifying the intracellular signaling pathways inducing Nur factors expression and their target genes is essential to elucidate their function in normal physiology as well as in addiction and anxiety disorders. These findings might offer novel targets to treat these devastating conditions.

### Conflict of interest statement

The authors declare that the research was conducted in the absence of any commercial or financial relationships that could be construed as a potential conflict of interest.
